# Predictors of depressive symptoms in patients with heart failure decompensation

**DOI:** 10.1192/j.eurpsy.2025.1261

**Published:** 2025-08-26

**Authors:** M. Zalewska, K. Nowak, T. Sanak

**Affiliations:** 1 Department of Emergency Medicine, Faculty of Health Sciences, Jagiellonian University Medical College; 2 Department of Coronary Artery Disease and Heart Failure, Jagiellonian University Medical College, John Paul II Hospital, Krakow, Poland

## Abstract

**Introduction:**

Acutely decompensated heart failure (HF) may be accompanied by depressive symptoms. Predictors of screening diagnosis of depression (SDD) in decompensated HF are poorly elucidated.

**Objectives:**

We sought to investigate independent determinants of SDD in patients with HF decompensation.

**Methods:**

Enrolled were fifty-one patients with a median age of 67 years and left ventricular ejection fraction (LVEF) of <40% hospitalized due to HF decompensation. SDD based on the result of the Beck Depression Inventory (>10 points), PHQ-9 (>12 points) or Hamilton Depression Scale (lack of depression: <7 points; mild [<12 points], moderate [<17 points] or severe [≥18 points] depression). Neurotrophic potential and response to stress were assessed by brain-derived neurotrophic factor (BDNF) and FK506 binding protein 5 (FKBP5), respectively.

**Results:**

SDD was identified in 26 (51%) patients based on the Beck inventory and in 12 (24%) patients based on PFQ-9. According to the Hamilton scale mild SDD was found in 17 (33.3%), moderate in 9 (17.6%) and severe in 14 (27.5%) of patients. Male patients with HF decompensation had higher result of Beck inventory by 35% (P=0.029), PHQ-9 by 60% (P=0.014) and Hamilton scale by 64% (P=0.003) than female. The result of the Beck Depression Inventory was correlated with plasma levels of FKBP5 (R=0.34, P=0.017) and inversely correlated with BDNF (R=-0.39, P=0.004). In turn, LVEF was correlated with the result of PHQ-9 (r=0.33, P=0.020), Hamilton scale (r=0.33, P=0.18) and with BDNF (r=0.32, P=0.025) while inversely with FKBP5 (r=-0.32, P=0.023). By multivariate analysis the higher result of the Beck inventory was associated with male gender (β=0.26, P=0.048), a higher LVEF (β=0.27, P=0.042) and a lower plasma BDNF (β=-0.46, P<0.001).

**Conclusions:**

SDD in patients with HF decompensation is independently associated with male gender, better preserved left ventricular systolic function and reduced plasma level of BDNF.

**Disclosure of Interest:**

None Declared

